# The role of inoculum and reactor configuration for microbial community composition and dynamics in mainstream partial nitritation anammox reactors

**DOI:** 10.1002/mbo3.456

**Published:** 2017-03-10

**Authors:** Shelesh Agrawal, Søren M. Karst, Eva M. Gilbert, Harald Horn, Per H. Nielsen, Susanne Lackner

**Affiliations:** ^1^ Technische Universität Darmstadt Institute IWAR Chair of Wastewater Engineering Darmstadt Germany; ^2^ Karlsruhe Institute of Technology Engler‐Bunte‐Institut Chair for Water Chemistry and Water Technology Karlsruhe Germany; ^3^ Center for Microbial Communities Department of Chemistry and Bioscience Aalborg University Aalborg Denmark; ^4^ EnviroChemie GmbH Rossdorf Germany

**Keywords:** biofilm, bioreactors, Molecular microbial ecology, Nitrogen metabolism

## Abstract

Implementation of partial nitritation anammox (PNA) in the mainstream (municipal wastewater treatment) is still under investigation. Microbial community structure and reactor type can influence the performance of PNA reactor; yet, little is known about the role of the community composition of the inoculum and the reactor configuration under mainstream conditions. Therefore, this study investigated the community structure of inocula of different origin and their consecutive community dynamics in four different lab‐scale PNA reactors with 16S rRNA gene amplicon sequencing. These reactors were operated for almost 1 year and subjected to realistic seasonal temperature fluctuations as in moderate climate regions, that is, from 20°C in summer to 10°C in winter. The sequencing analysis revealed that the bacterial community in the reactors comprised: (1) a nitrifying community (consisting of anaerobic ammonium‐oxidizing bacteria (AnAOB), ammonia‐oxidizing bacteria (AOB), and nitrite‐oxidizing bacteria (NOB)); (2) different heterotrophic denitrifying bacteria and other putative heterotrophic bacteria (HB). The nitrifying community was the same in all four reactors at the genus level, although the biomasses were of different origin. Community dynamics revealed a stable community in the moving bed biofilm reactors (MBBR) in contrast to the sequencing batch reactors (SBR) at the genus level. Moreover, the reactor design seemed to influence the community dynamics, and reactor operation significantly influenced the overall community composition. The MBBR seems to be the reactor type of choice for mainstream wastewater treatment.

## 1 Introduction

Partial nitritation anammox (PNA) is the combination of anaerobic ammonium oxidation (Anammox) with partial nitrification. Implementation of PNA for mainstream (municipal) wastewater treatment (WWT) can result in a paradigm shift for municipal WWT, as it holds promise to lower energy demands, enables better use of the organic carbon, and saves costs for excess sludge management (Lackner et al., 2015; Siegrist, Salzgeber, Eugster, & Joss, 2008). However, its applicability to the mainstream is still unclear and under investigation. PNA is a complex biotechnological application where controlled partial nitrification (nitritation) through aerobic ammonia‐oxidizing bacteria (AOB) is required to provide an ideal ratio of 1.32 g NO^‐^_2_‐N/g NH^+^_4_‐N, for anammox activity under anoxic conditions (Strous, Kuenen, & Jetten, 1999). Maintaining these conditions is challenging due to an intrinsic competitive microbial environment. To improve process understanding for better reactor performances and process stability, a link to microbial ecology is essential (Rittmann, 2006).

Several studies have investigated the microbial community in PNA systems. The key members of this microbial community are AOB and anaerobic ammonium‐oxidizing bacteria (AnAOB), and nitrite‐oxidizing bacteria (NOB) (Park, Rosenthal, Ramalingam, Fillos, & Chandran, 2010; Persson et al., 2014; Tsushima, Kindaichi, & Okabe, 2007). Some studies also reported putative heterotrophic denitrifying bacteria (HB) in PNA systems based on clone libraries (Gilbert et al., 2014; Langone et al., 2014; Park, Rosenthal, Ramalingam, et al. 2010).

Despite the importance of HB for the PNA process—positive (Desloover et al., 2011; Wang et al., 2010) or negative (Bürgmann, Jenni, Vazquez, & Udert, 2011; Lackner, Terada, & Smets, 2008)—characterization of the heterotrophic bacteria has been very limited, until recently. Only a few studies have provided detailed insight into the community composition of the PNA systems using next‐generation sequencing platforms (Chu et al., 2015; Costa et al., 2014; Pellicer‐Nácher et al., 2010; Pereira et al., 2014; Speth, in ‘t Zandt, Guerrero‐Cruz, Dutilh, & Jetten, 2016). These studies reported two important aspects: (1) a vastly diverse community composition in PNA systems, (2) the need to further investigate the whole community composition and structure in PNA systems, to shed more light on the role of each community member for removing nitrogen in PNA systems, directly or indirectly. However, these studies were performed on systems which were not operated under mainstream conditions, rather as anammox enrichment reactors or sidestream PNA reactors. Thus, knowledge about whole microbial community structure of PNA systems under mainstream conditions remains to be elucidated.

For start‐up of sidestream PNA systems, it is common practice to inoculate with biomass from existing PNA systems due to the low specific growth rates of AnAOB. Such a start‐up strategy is even more important for mainstream PNA systems due to low temperatures and low nitrogen concentrations and thus even lower specific activities and growth rates of both AnAOB and AOB (Hendrickx et al., 2012). However, previous studies have reported that the PNA reactors contain only one dominant AnAOB (Hu et al., 2010; Park, Rosenthal, Jezek, et al. 2010), indicating that specific environmental conditions might support one AnAOB over another. For example, anammox reactors with continuously high nitrite concentrations and COD: N ratios reported a predominance of the genus *Ca*. Brocadia over other AnAOB phenotypes (Jenni, Vlaeminck, Morgenroth, & Udert, 2014; Laureni et al., 2015). There is, however, no clear consensus in previous studies on what drives the development of one particular anammox strain over another. Such variations also extend to the occurrence of different genera of AOB. Therefore, selection of the right inoculum for a mainstream PNA system might be crucial due to variations in the dominant AnAOB and AOB. The choice of the right inoculum might extend to other community members too, as they are also significant (Speth et al., 2016).

All known AnAOB genera have been identified in different PNA system configurations (Egli et al., 2001; Park, Rosenthal, Jezek, et al. 2010; Park, Rosenthal, Ramalingam, et al. 2010; van der Star et al., 2007; Wang et al., 2010). A recent study highlighted the need for a deep taxonomic resolution, that is, down to genus or species level. They reported that the biomass containing *Ca*. Brocadia fulgida seemed to be less influenced by a decrease in temperature than biomass containing *Ca*. Brocadia sinica (Lotti, Kleerebezem, & van Loosdrecht, 2015). Other studies reported biofilm PNA systems with *Ca*. Brocadia as a preferred choice at low temperature (Gilbert, Agrawal, Schwartz, Horn, & Lackner, 2015; Lackner & Horn, 2013; Laureni et al., 2016). Fast start‐up and stable performance of mainstream PNA systems thus relies on the inoculum composition and the reactor configuration. 16S rRNA gene amplicon sequencing provides such a resolution for comprehensive community determination, although it also has its pitfalls (Albertsen, Karst, Ziegler, Kirkegaard, & Nielsen, 2015).

The question remains how different the biomass composition in different full‐scale PNA systems actually is, and thus, how important it is to choose the “right” inoculum considering that the microbial community structure might be distinct for different wastewater compositions, plant configurations, and operating conditions. Also, the subsequent impact of a temperature decrease and the adjusted implementation of PNA in the mainstreams are still unclear.

In our previous study (Gilbert et al., 2015), we compared process performance of four different PNA lab‐scale reactors operated under mainstream conditions. The biomass originated from four different full‐scale PNA reactors at three different locations. We also monitored the abundances of AnAOB, AOB, and NOB based on the qPCR analysis. The qPCR results suggested the presence of other abundant microbial members. Also, the process performance data revealed significant differences in performance among the four lab‐reactors . These findings raised further concerns on: (1) the analysis of the whole microbial community and its composition, (2) differences in the community composition among the inocula and the responsibility for differences in reactor performances.

Therefore, in this study, we focused on answering the questions based on findings from other studies as well as our previous work. The overall aim of this study was twofold: (1) a comprehensive comparison of the microbial community composition of the biomasses from four full‐scale sidestream PNA systems which were used as inocula in the four lab‐scale reactors operated under mainstream conditions; (2) in‐depth monitoring of the species abundance distribution and their dynamics in these lab‐scale reactors, subjected to a gradual temperature decrease and low nitrogen concentrations, and determine the correlation between the previously reported reactor performances with the response of the whole community to the change in conditions.

## 2 Materials and Methods

### 2.1 Reactor setup and operation

Our model systems, to gain comprehensive insight into the composition and dynamics of the total microbial community of the four different PNA biomasses, were four 10 l lab‐scale reactors (Gilbert et al., 2015)—two sequencing batch reactors (SBRs), one with suspended biomass (SBR1) and one with granular biomass (SBR2); two moving bed biofilm reactors (MBBRs), one with BiofilmChip^™^ M carrier material (MBBR1) and one with K3^®^ carrier material (MBBR2), (both AnoxKaldnes AB, Lund, Sweden) which were operated as described in Gilbert et al. (2015). These reactors were of particular interest, as the biomass in these reactors originated from four different full‐scale sidestream PNA which also differ in their biomass enrichment methods (Table 1), and their biomass is used to inoculate other PNA reactors in Europe. All four lab‐reactors were operated in parallel and fed with synthetic wastewater with 50 mg‐N l^−1^ ammonium (see also Suppl. Information). The reactors were equipped with online sensors for dissolved oxygen (DO), temperature, pH, conductivity, and ammonium and nitrate (Endress+Hauser, Germany). All reactors were controlled based on ammonium effluent concentrations which were set to 6–8 mg‐N l^−1^ by adjusting the influent flow rate. The pH was maintained at 7.3 ± 0.3, DO and biomass concentrations are provided in Table 1. Reactor performances and microbial population dynamics were monitored over a period of 45 weeks. The applied temperature profile is shown in Fig. S1 and was as follows: reactor operation started at week 0 and during phase I, the reactors were operated at 20°C (15 weeks); in phase II, the temperature was gradually decreased by 0.5°C every 7 days down to a temperature of 10°C (weeks 16–35); phase III covered operation at constant 10°C (week 36–45).

**Table 1:** Characterization of the inocula and reactor operation data (averages over the entire 45 weeks)

		SBR 1	SBR 2	MBBR 1	MBBR 2
Initial biomass					
Biomass origin		Sidestream SBR, Germany	Sidestream IC reactor the Netherlands^a^	Sidestream MBBR Sweden	Sidestream MBBR Sweden
Inoculation		from sidestream SBR	none	Sweden from pilot plant/effluent from sidestream MBBR	
Biomass type		Suspended (x_90,3_ = 640 μm)	Granulated (x_90,3_ = 2070 μm)	Biofilm (max. thickness 2 mm)	Biofilm (max. thickens 10 mm)
lab‐reactors					
TSS [g l^−1^]		0.8 ± 0.5	2.0 ± 0.5	5.7 ± 0.5	9.3 ± 0.8
VSS [%]		60 ± 5	56 ± 0.3	68 ± 2	70 ± 1.5
Average DO [mg l^−1^]		0.09 ± 0.15	0.11 ± 0.08	0.20 ± 0.17	0.25 ± 0.13
HRT [d]		6.3 ± 4.8	2.6 ± 1.3	1.4 ± 0.8	1.5 ± 0.9

x_90,3_, particle size from Feret diameter; TSS, total suspended solid; VSS, volatile suspended solids; HRT, hydraulic retention time; DO, dissolved oxygen.

a IC reactor, Internal circulation reactor.

### 2.2 Microbial community analysis

#### 2.2.1 Sampling and amplicon library preparation

Biomass samples were collected from the reactors during operation and stored at −80°C for further analysis (see also Suppl. Information). Extracted genomic DNA was used for library preparation employing a procedure adapted from Caporaso et al. (2011). Using 5 ng/μl of DNA, template PCR amplification (see also Suppl. Information) was performed in duplicate using hypervariable region V4 primers (targeting 253 bp partial 16S rRNA gene sequence) fused with barcodes and adapters for the Illumina MiSeq platform. The duplicate library reactions were pooled together, and the libraries were cleaned using Agencourt AMPure XP beads (Beckman Coulter) with the standard protocol. The quality of the library was checked using TapeStation and D1K ScreenTape (Agilent Technologies Inc., USA).

#### 2.2.2 Sequencing and sequence analysis

The multiplexed amplicon library was sequenced on a MiSeq System (Illumina) using MiSeq Reagent Kit v3 (Illumina). Samples from the libraries were analyzed by a quick quality check to evaluate overall data quality (check the quality score, the occurrence of ambiguous bases and k‐mer abundances). Afterward, the libraries were processed to (1) truncate read 1 to 250 bp length and discard read 2; (2) screen for PhiX contamination; (3) format metadata for QIIME; and (4) remove unique reads and normalize libraries to an even depth of 10,000 reads per sample.

OTU (operational taxonomical unit) picking and classification was done using QIIME v. 1.7 (quantitative insight into microbial ecology) (Caporaso et al., 2010) with its default settings. Overall, the de novo clustering of OTUs was done with 97% identity, corresponding to species level. The sequences were then classified using the RDP (Ribosomal Database Project) classifier (80% confidence threshold) based on the taxonomy in the Greengenes database (97% confidence threshold, version 13.5 May 2013) (McDonald et al., 2012). Additionally, NCBI BLAST database was used to determine the closest bacterial relatives (having 100% sequence homology) of the reported OTUs. OTU representative sequences have been submitted to the GenBank under the accession numbers KY226724 ‐ KY228337.

#### 2.2.3 Data analysis

The sequencing data was analyzed using R to gain insight into the overall community structure dynamics over time in all four reactors individually. To analyze the core representatives of the microbial community, OTU count data from QIIME was imported into R in biom format along with a mapping file containing sample metadata. Key R‐packages used for analysis were phyloseq (v1.7.12) (McMurdie & Holmes, 2013), ggplot2 (v0.9.3.1), and VEGAN (v 2.0.9). The Phyloseq package was used considering its function as a wrapper for functions from other ecological packages including VEGAN. The Alpha and Beta diversity indices of the samples were calculated from the normalized data. Community dynamics comparison within the reactors corresponding to specific taxa was performed with constrained correspondence analysis (CCA). Square‐root transformed and centered OTU counts were used for constrained CCA analysis. Data were constrained by temperature, as lowering of temperature was the variable parameter in the experiment**.**

## 3 Results and Discussion

### 3.1 Characterization of the inocula—microbial community composition

The biomasses that were used to inoculate the four lab‐scale reactors, without any cross‐inoculation during the 45 weeks of operation, originated from different full‐scale sidestream facilities treating centrates from sludge dewatering units. The operating conditions at these sites differed with influent ammonium concentrations of 500–1,000 mg‐N l^−1^, effluent ammonium concentrations around 50–100 mg‐N l^−1^, and operating temperatures between 22 and 34°C, caused by the installation in the sidestream (after anaerobic digestion) (Lackner et al., 2014).

The characteristics of the biomasses are summarized in Table 1. The biomasses varied in their physical structure (type) as a result of the reactor type: two inocula were from sequencing batch reactors, suspended (SBR1) and granulated (SBR2) biomasses, respectively, characterized by different particle size distributions. The other two inocula came on carrier materials with biofilms of maximum thicknesses of 2 mm (MBBR1) and 10 mm (MBBR2), respectively. For clarity, in this study, the total microbial community was divided into a nitrifying microbial community (NMC, typically studied community) and a heterotrophic microbial community (HMC). The NMC included AOB, AnAOB, and NOB; the HMC included putative heterotrophic bacteria, (partial) denitrifiers (HB), and denitrification intermediate reducers (only reducing nitrogen oxides).

**Figure 1:**
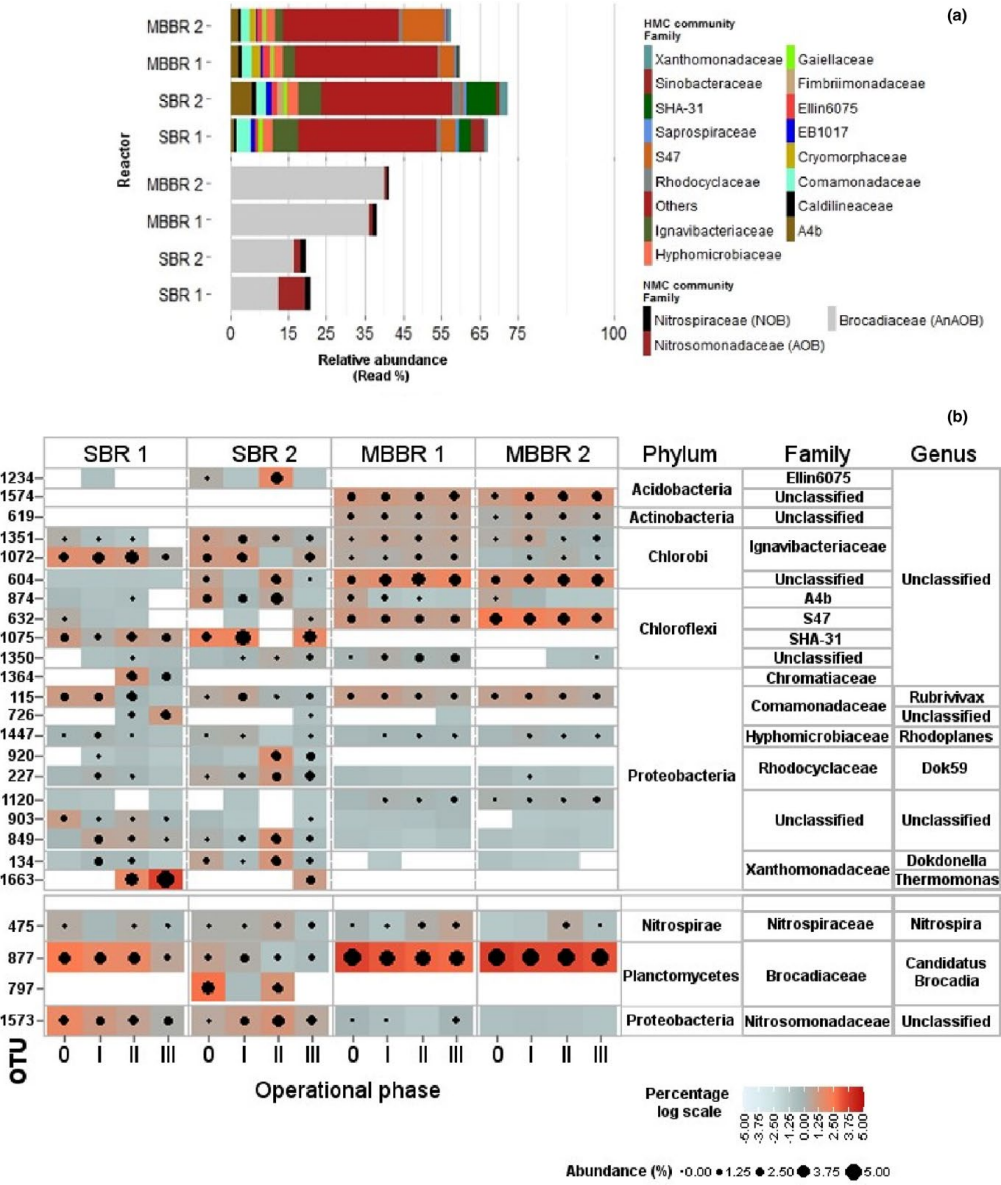
(a) Microbial community composition of the biomasses from four full‐scale PNA plants (used as inoculum for each lab‐reactor) at family level. For heterotrophic microbial community (HMC), families contributing <2% to the total relative read abundance are merged as others. (b) Microbial community composition and dynamics at OTU level. This heat map shows the relative abundance of the most dominant microbial population in each reactor. The top 10 OTUs from each reactor were selected (a total of 25 OTUs from all the samples). The associated phyla, families, and genera are on the right panel of the heat map. Upper panel shows OTUs associated with putative heterotrophs and lower panel for the nitrifying community. Color Key (natural log scale of relative percentage read abundance) shows the related abundance in each sample. Point scale (natural log scale of relative percentage read abundance) presents the OTUs with =<1% of the read abundance. SBR1 (suspended biomass), SBR2 (granular biomass), MBBR1 (BiofilmChip^™^ M), and MBBR2 (K3^®^)

All four inocula had similar NMC diversity, constituting similar family members of the phyla Planctomycetes (AnAOB), Nitrospirae (NOB), and Proteobacteria (AOB). In all inocula, the AnAOB belonged to the genus *Ca*. Brocadia, the AOBs to unclassified members of the family Nitrosomonadaceae, and the NOB to the genus *Nitrospira*; only the relative read abundance differed (Figure 1). The classification of AOB was limited to the family Nitrosomonadaceae. Therefore, manual search with other databases (RDP classifier and NCBI) was performed. The closest relative identity belonged to the *Nitrosomonas europaea‐eutropha* group. In the MBBRs, AnAOB accounted for nearly 35%–40%, AOB for ~1%, and NOB <1%; in the SBRs, the fraction of AnAOB was 12%–20%, AOB 1%–3%, and NOB 2%–5%. Also within the HMC, the same dominant phyla were present in all the inocula (Fig. S2), dominated by similar family members of the phyla Proteobacteria, Chloroflexi, and Chlorobi. The relative read abundances of this group (HMC) varied mainly between SBRs and MBBRs, with an overall percentage of 55% to 75% of the total relative abundances (Figure 1a, Fig. S2).

Similarities between the inocula were also found on the OTU species level, although the inocula had such different origins. Especially within the NMC, all the inocula shared a single dominant OTU for each NMC member (Figure 1b). Only the inoculum of SBR2 had an additional dominant OTU within the AnAOB. The closest relative to OTU 877 found in databases was *Ca*. Brocadia sp. 40 and *Ca*. Brocadia sinica JPN1 to OTU 797. Aside from the NMC, the presence/occurrence of the common dominant OTUs within the HMC was also evaluated. The top 10 dominant OTUs were selected from each reactor, and common OTUs were compared and then distinguished based on the following three categories: (1) shared by all reactors, (2) shared among SBRs and MBBRs, respectively, and (3) common in any three reactors (Figure 1). Similar trends were observed even for the whole community composition at OTU level: all four inocula had the largest overlap followed by the overlap within the MBBRs and SBRs, respectively (Fig.S3, S.Fig. S4A). The dominant OTUs common in all four inocula were affiliated to the genera *Rubrivivax*,* Rhodoplanes*, Dok59, unclassified members of the families A4b, and Ignavibacteriaceae (Figure 1b). The dominant OTUs affiliated to SHA‐31, Xanthomonadaceae, and some unclassified families were present only in the SBRs; some OTUs affiliated to unclassified families were specific to the MBBRs only. This observed differentiation between the SBRs and MBBRs was further confirmed by the richness diversity indexes (Table S2) and PCA analysis (See Fig. S3 for details).

The results clearly showed that the predominant NMC members and several HMC members were present in all the inocula, which was not expected considering the different settings (reactor type, wastewater composition) at each site. Similar to our result characterizing the inocula, other studies also reported low fractions of AOB and NOB in PNA reactors (Almstrand et al., 2014; Kindaichi et al., 2007). The relative abundance of *Ca*. Brocadia was relatively high in all four inocula, but it was the HMC that constituted the major biomass fraction of each inoculum (Figure 1a) although the COD/N ratios were <2 in the systems of origin (Lackner et al., 2014). Our results are in congruence with previous studies about the coexistence of HB in anammox systems (Costa et al., 2014; Garcia Costas et al., 2012; Kindaichi et al., 2007; Langone et al., 2014; Laureni et al., 2015; Ni, Ruscalleda, & Smets, 2012; Speth et al., 2016). In a study (Ni et al., 2012), the presence of (residual) organic content including soluble microbial products (SMP) in the side‐ and mainstream wastewaters was reported to support the HMC in PNA systems.

In our study, *Ca*. Brocadia was the single identical AnAOB dominant in all reactors, which deviates from research that detected eight unique dominant AnAOB phylotypes in eight different nitrogen‐removal reactors (Hu et al., 2010). The genus *Ca*. Brocadia had a single dominant OTU in all the inocula except SBR2, which had one additional OTU. *Ca*. Brocadia sp. 40 and *Ca*. Brocadia sinica, closest related to OTU 877 and OTU 797, respectively, have been identified to thrive in NH_4_^+^ and NO_2_^‐^ rich environments (Laureni et al., 2015; Oshiki, Satoh, & Okabe, 2016; Oshiki, Shimokawa, Fujii, Satoh, & Okabe, 2011; Park, Rosenthal, Ramalingam, et al. 2010). The reason for detecting these OTUs in our reactors was that the biomasses originated from PNA systems treating wastewaters high in nitrogen concentration.

Interestingly, all biomasses fostered the same AOB and NOB, sharing a single common OTU related to *Nitrosomonas europaea‐eutropha* and *Nitrospira,* respectively. These observations can be explained based on the aeration strategies employed in PNA systems—limited oxygen, and therefore, lower transient nitrite availability. These conditions are reported to support *Nitrosomonas europaea‐eutropha* (Li et al., 2009) and *Nitrospira* (Park, Rosenthal, Jezek, et al. 2010).

These results suggest that very similar NMC compositions were obtained in PNA systems if operated under generally similar conditions, and irrespective of reactor configuration. Our results are in partial agreement with another study (Park, Rosenthal, Jezek, et al. 2010), in which different reactor types (i.e., SBR and MBBR inoculated with different biomasses) constituted *Ca*. Brocadia sp. 40 but different *Nitrosomonas* species. A recent study (Laureni et al., 2015) also emphasized the presence of *Ca*. Brocadia in mainstream PNA systems exposed to complex substrates over other anammox species.

Furthermore, our results disclosed the coexistence of dominant OTUs of the families Comamonadaceae, Hyphomicrobiaceae, Rhodocyclaceae, A4b, Sinobacteraceae, Xanthomonadaceae, and Ignavibacteriaceae in all the inocula, although with diverse abundances, as also reported in previous studies (Kindaichi, Yuri, Ozaki, & Ohashi, 2012). Presumably, these HB can coexist in different PNA systems due to the existence of variable macro‐ and microenvironments (Speth et al., 2016). The high abundance and similarity of the HMC highlight that not only does NMC belong to the core community in PNA systems but also the HMC members. The different oxic and anoxic phases, and the presence of substrate gradients due to biomass structure provide conditions for such a diverse core community.

Based on the HMC members detected, the biomasses are metabolically very versatile, comprising members which can carry out (partial) denitrification, metabolize organic carbon (an) aerobically, and assist in forming microbial aggregates. The families Comamonadaceae, Hyphomicrobiaceae, Rhodocyclaceae, Sinobacteraceae, Ignavibacteriaceae, and SJA‐28 contain known (partial) denitrifiers and/or denitrification intermediate reducers (Liu et al., 2012; Ni et al., 2011; Sadaie et al., 2007). Chloroflexi was the dominant filamentous species, known to support aggregate formation (Ni et al., 2011), and reported to scavenge secondary metabolites derived from AnAOB under anoxic conditions (Kindaichi et al., 2012). Some of them can also denitrify (McIlroy et al., 2016). Such high diversity and functional redundancy indicate their importance in the PNA process.

### 3.2 Reactor performance

Detailed performance analyses of all four reactors have been reported previously (Gilbert et al., 2015). Here, we summarize the operational performance of these reactors and determine reasons for the observed performance change in conjunction with the response of the whole microbial community to mainstream conditions. With the start of reactor operation (week 0), the temperature was set to 20°C and only ammonium and nutrients were fed to the lab‐reactors, hence removing any external carbon source, compared to the conditions at the full‐scale facilities (a few hundred mg/L organic carbon measured as COD). These reactors were operated with organic carbon‐free influent to reduce the potential contribution of denitrification to PNA and to be able to attribute changes mainly to the temperature gradient. All four lab‐reactors were exposed to the same temperature profile over three defined phases (Fig. S1)—phase I (weeks 0–15), start‐up at 20°C; phase II (weeks 16–35), temperature gradient (20°C to 10°C); and phase III (weeks 36–45) operation at 10°C. Table 1 provides the main operating parameters. Over the course of this study, biomass concentrations were quite stable. However, the concentrations differed significantly between the reactors, with 0.8 and 2.0 g‐TSS l^−1^ for the SBRs, and 5.7 and 9.3 g‐TSS l^−1^ for the MBBRs (Table 1). Deviations in operational parameters (HRT and DO) were based on the biomass characteristics, that is, different thicknesses and total amounts.

**Figure 2:**
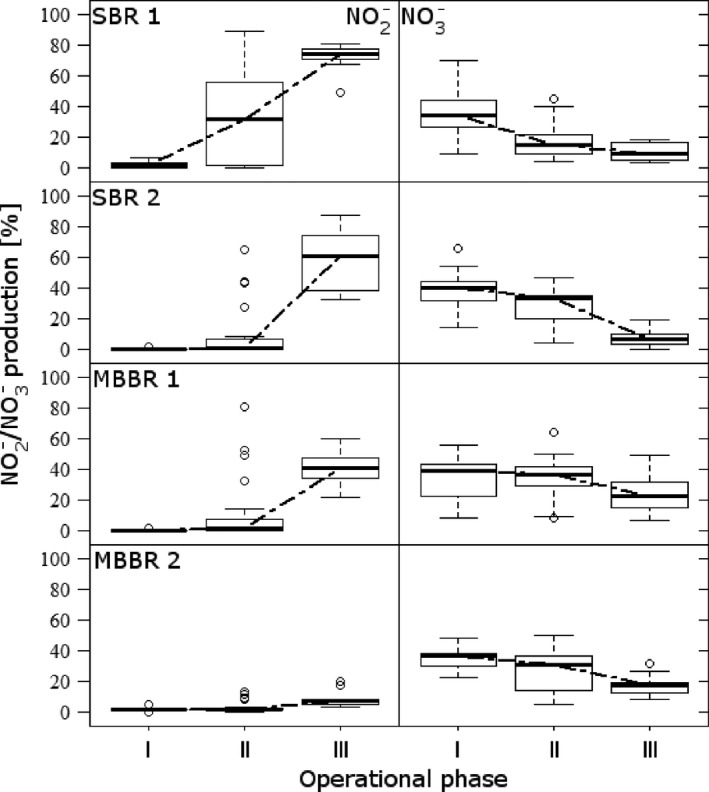
Reactor performance (average over complete dataset during each phase) during the three phases: I – 20°C, II – 20°C–10°C, III 10°C, expressed as nitrite (left column) and nitrate (right column) produced, based on the ammonium converted, in all four reactors. Box plots show median values (horizontal line), as well as 25th and 75th percentiles (box) and 10th and 90th percentiles (errors bars)

Reactor performances are summarized in Figure 2 with additional details in S. Table 1. Initial nitrogen loadings (week 0) were highest in the MBBRs (around 200 g‐N m^−3^ d^−1^), followed by SBR2 (approx. 80 g‐N m^−3^ d^−1^) and SBR1 (40 g‐N m^−3^ d^−1^), which was mainly attributed to the different biomass concentrations. The ammonium removal was above 90% and similar in all four reactors. By the end of phase I, the nitrogen loading was reduced to maintain the effluent concentration. The biomass‐specific conversion rates were between 5 and 8 g‐N kg‐TSS^−1^ d^−1^. During the temperature decrease (phase II), the turnover rate reduced by a factor of 2, as was expected for a temperature decrease down to 10°C. In phase III, ammonium removal remained rather stable.

Nitrite and nitrate production significantly differed between the reactors. During phase II, a significant nitrite accumulation occurred in SBR1 and SBR2 (from almost 0% (week 15) to 62% in SBR1 and 54% in SBR2 (week 35)), with a simultaneous decrease in nitrate production to almost nothing (3%–6% in week 35) (Figure 2, Table S1). Nitrite accumulation in MBBR1 also increased significantly from 0% to 30% in phase III. Only MBBR2 did not accumulate any nitrite during the temperature decrease. In phase III, nitrite production remained at high levels except for MBBR2 (<5% of the converted ammonium). Nitrate production decreased in all reactors during the temperature gradient from 20°C to 10°C from 25%–40% to less than 15%. The reactor performances correlated well with biomass structure and aggregate/biofilm thickness. MBBR2 with a biofilm of up to 10 mm thickness performed best (least nitrite and nitrate accumulation), whereas the reactors with thinner biofilms/smaller aggregates struggled with nitrite accumulation.

### 3.3 Microbial community dynamics

**Figure 3:**
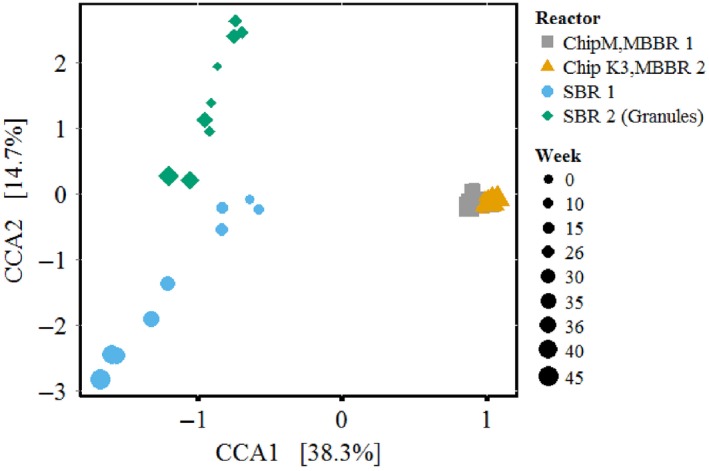
Constrained correspondence analysis plot for all four reactors over time, exhibiting the influence of the change in operating temperature (i.e., 20°C to 10°C). SBR1 (suspended biomass), SBR2 (granular biomass), MBBR1 (BiofilmChipTM M), and MBBR2 (K3^®^). The *x*‐axis (horizontal) refers to differences between reactor types. The *y*‐axis (vertical) relates to the change in temperature/time

The results of the reactor operation suggest that the systems were influenced differently by the temperature gradient, although influent composition and operating conditions were the same in all four reactors. The question was now whether the microbial community composition could explain the differences in behavior. Therefore, a constrained correspondence analysis (CCA) was performed as a first step to ascertain whether the temperature change affected the community composition over time. This analysis revealed that the microbial community composition in the MBBRs was relatively stable, whereas it was highly dynamic in the SBRs (Figure 3). The response of the community composition showed a high correlation (ANOVA (analysis of variance), *p* < .001) to the temperature decrease and nitrite accumulation (Figure 2, Figure 3). The influence of the temperature change on community structure was more significant for the SBRs compared to the MBBRs, especially in SBR1, which exhibited a continuous instability during the entire operation, corresponding to lower efficiency (Figure 2, and Supplementary information for details).

#### 3.3.1 Nitrifying microbial community

**Figure 4:**
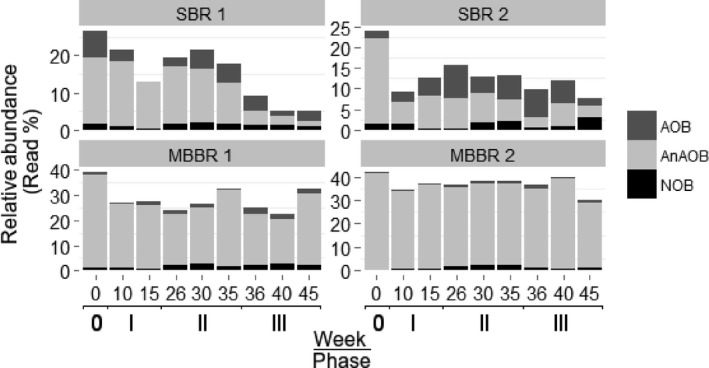
Relative abundance of nitrifying microorganisms in all the reactors. SBR1 (suspended biomass), SBR2 (granular biomass), MBBR1 (2 mm biofilm), and MBBR2 (10 mm biofilm)

The correlation of the community dynamics with the nitrite buildup suggested a significant influence of the temperature decrease on the Nitrifying microbial community (NMC). However, nitrite buildup can originate from loss of AnAOB activity as well as from loss of NOB activity or a relative increase in AOB activity; thus, causing overproduction of nitrite. A closer look at the individual members of the NMC might help to better understand the exact circumstances. The relative abundances, especially of AOB and *Ca*. Brocadia (AnAOB), decreased in all four reactors in week 10 (phase I) compared to the respective inocula (Figure 1B, Figure 4, Fig. S6). Afterward, *Ca*. Brocadia in the MBBRs (represented by OTU 877) was quite stable with a sequence read fraction significantly higher than in the SBRs (Figure 1b, Figure 4). The relative read abundances in the SBRs were less stable and decreased over time. In SBR1, the relative abundance of *Ca*. Brocadia reduced to <5% in week 36 (phase III) and further to 1.2% at the end of phase III (week 45, 10°C); in SBR2, it reduced to ~3% in week 36 (phase III), which coincided with the absence of another OTU (*Ca*. Brocadia, OTU 797). No change in the relative abundance of AOB was observed in the MBBRs; minor changes appeared in the SBRs. AOB abundance was low and represented by the single OTU 1573 in all the reactors over the entire time. The NOBs, also represented by a single OTU (*Nitrospira*, OTU 475) (Figure 1b, Figure 4), exhibited a change in the relative abundance also in the MBBRs, though it was only minor; OTU 475 increased to 1.5% during phase II in all reactors and remained stable except in MBBR2 (reduced again in phase III). Overall, the loss in *Ca*. Brocadia abundance, especially in the SBRs, seemed to be the most influential part for the buildup of nitrite.

#### 3.3.2 Heterotrophic microbial community

Another reason for the differences in reactor performances might stem from the large Heterotrophic microbial community (HMC) in all reactors. Diversity and richness within the HMC at the end of phase I increased in all reactors except SBR1 compared to the inocula biomasses (Fig. S6, Table S2). Afterward, the differentiation in community dynamics among the SBRs and MBBRs was analogous to the NMC (Fig. S2, Fig. S6). The community diversity in the MBBRs was relatively stable compared to the SBRs. The relative abundance profiles of the most dominating OTUs associated with different phyla within the HMC differed in the four reactors (Figure 1b, Table S3). They belonged to the genera *Rubrivivax*,* Rhodoplanes*, Dok59, *Dokdonella*,* Thermomonas*, and some unclassified members of the phyla Chloroflexi, Chlorobi, Actinobacteria, and Acidobacteria. Few OTUs in the MBBRs and SBRs behaved similarly in the different phases (Table S3). During phase II, the relative abundance of some OTUs increased significantly in the SBRs (Figure 1b, Fig. S7). For instance, *Thermomonas* (OTU 1663) increased, especially in SBR1, and this OTU accounted for up to 60% of the total relative read abundance. Several other OTUs also showed specific responses, for example, the relative abundance within the families Comamonadaceae (OTU 726) and Chromatiaceae (OTU 1364) increased (up to 10%) in SBR1; OTU 920 and OTU 227 from genus Dok59 increased (up to 9%) in SBR2. No significant change occurred in the MBBRs during phase II. In phase III, the abundance profiles of various OTUs associated to Chloroflexi (Anaerolineae class) reduced in all the reactors. Additionally, the abundance of Chlorobi (OTU 1072) decreased to a fourth in SBR1; however, it increased in MBBRs.

### 3.4 Interrelationship between microbial community, reactor type, and reactor performance

This study clearly showed that the temporal community dynamics were linked to the reactor type and driven by a temperature decrease. The communities shifted in all reactors from the initial inocula (Fig. S5), particularly the abundance of the nitrifying community reduced, attributed to the selective pressure imposed by changes in the operational conditions. Although the inocula of all four lab‐reactors harbored *Ca*. Brocadia (as the same AnAOB) and the same AOB at OTU species level, the response to the temperature change varied significantly. The imposed temperature decrease led to a significant reduction in the relative abundance of *Ca*. Brocadia in the SBRs compared to the MBBRs, which corresponded to the different volumetric ammonium removal rates at 10°C (approx. 5–8 g‐N m^−3^ d^−1^ in the SBRs and 12–16 g‐N m^−3^ d^−1^ in the MBBRs). These rates were similar to other studies (Dosta et al., 2008; Hu et al., 2013). MBBR2 performed better than the other reactors, especially in terms of stability (lowest nitrite accumulation). This suggests that the reactor configuration is an important factor. In this study, biofilm thickness differed significantly with better performances for thicker biofilms/aggregates.

Despite great interest in mainstream PNA and availability of many different biomasses and reactor types for bio‐augmentation, efforts to evaluate the temperature effect on various biomasses is still very limited. Recently, a study evaluated the behavior of different biomasses at a lower temperature by batch experiments, however, restricted to AnAOB and AOB (Lotti et al., 2015). They reported that the AnAOB activity in biofilms was less influenced than in suspended biomass. We found a strong correlation between the NMC composition and the change in temperature, the nitrite accumulation (i.e., performance and stability), and the reactor type in our long‐term study. Interestingly, the observed NMC dynamics occurred only within the framework of the initially detected OTUs. The magnitude of the effect of the temperature decrease was linked to the thickness of the microbial aggregates. In our study, SBR1 (with suspended biomass) responded first by a decrease in AnAOB population and sudden nitrite accumulation, followed by SBR2 (granular biomass). The biofilm system of MBBR1 (thin biofilm) showed a similar response to nitrite accumulation although the AnAOB abundance was stable. Only MBBR2 maintained a stable performance. This suggests that temperature influenced abundance and activity. Considering these reactors were fed only with ammonium, the source for nitrite can be either ammonium oxidation by AOB and/or reduction of available nitrate to nitrite by partial denitrifiers, whereas the nitrite sink can be anaerobic ammonium oxidation by AnAOB, nitrite oxidation by NOB, or nitrite reduction by nitrite reducers. The continuous presence of nitrite in Phase II and III suggests an imbalance between nitrite source and sink. AOB were producing nitrite, but its consumption was not counterbalanced by either of the microbial members responsible for the nitrite sink. AnAOB were influenced by the temperature decrease rather than the competition for nitrite imposed by NOB and denitrifiers, which is often attributed to the failure of PNA systems (Joss et al., 2011; Lackner et al., 2015). Moreover, NOB also seemed to be negatively influenced by the temperature decrease. Although nitrite was available, no increase in their population was observed. Huang, Gedalanga, Asvapathanagul, and Olson (2010) also reported negative impact of low temperature on *Nitrospira* (also detected in our study). The accumulation of NO_2_^‐^ (Figure 2) clearly indicated that AOB activity and competition for nitrite were not responsible for the poor reactor performance below 13°C.

The absence of organic carbon in the influent of the lab‐reactors had no significant influence on the composition of the HMC. Thus, in consensus with previous studies (Ni et al., 2011, 2012), most likely, soluble microbial products from the NMC provided the carbon source for the HMC members. Temperature and nitrite concentrations influenced the dynamics of the HMC. For instance, nitrite accumulation during phase II led to an increase of OTUs associated with Chlorobi (Ignavibacteriaceae), Xanthomonadaceae, Comamonadaceae, Rhodocyclaceae, and Chromatiaceae, SJA‐28 (Figure 1b, Fig. S7, Table S4), respectively. Such a response might have been caused by (1) high nitrite availability that triggered direct nitrite reduction without relying on nitrate availability. For example, Xanthomonadaceae members only reduce nitrite to nitrous oxide (N_2_O) (Finkmann, Altendorf, Stackebrandt, & Lipski, 2000). (2) Increase in growth of one community member support growth of others, as most members in PNA biomass rely on exchange and transfer of nitrogen cycle intermediates among each other (Speth et al., 2016). At 10°C, temperature played an important role in the composition of the HMC. Except Xanthomonadaceae (OTU 1663, a closest relative was identified as *Thermomonas fusca,* which can thrive at 10°C (Mergaert, Cnockaert, & Swings, 2003; Langone et al., 2014)) other members of the HMC were negatively affected. This observation is supported by previous studies, that lower temperature has a negative impact on denitrifiers (Lu, Chandran, & Stensel, 2014). Additionally, the reactor configuration was also relevant for the dynamics of the HMC. The MBBRs had a rather stable total community composition compared to the SBRs. Similar results were obtained in a study by Laureni et al. (2016), who reported higher community dynamics in suspended biomass compared to biofilm carriers in PNA reactors operated at 15°C. Such community behavior seemed to be caused by the lack of structural preservation which was available in the MBBRs.

## 4 Conclusions

The detailed microbial community study performed on biomasses originating from different full‐scale PNA sidestream systems (as potential inocula for mainstream application) and their response to mainstream conditions in the four lab‐scale reactors led to the following key conclusions: (1) Differently configured PNA systems operated under generally similar conditions revealed a similar microbial community composition of the functional microbial community (i.e., AOB, AnAOB), including the NOB and putative heterotrophs (including (partial) denitrifiers a.o.). Thus, more studies are required to determine the PNA core community. (2) Ubiquitous presence of the HMC in all lab‐reactors treating synthetic feed free of organic carbon underlined their significance for nitrogen removal. Further research to understand this intricate community and its correlation with the mainstream PNA systems in the presence of external organic carbon along with soluble microbial products, that is, the influence of organic carbon on the HMC composition and its role in PNA systems, should be the focus of future research. (3) The impact of temperature decrease on the performance and comprehensive community composition in PNA systems under mainstream conditions correlated with the reactor configuration. The MBBRs showed better performance and a more stable operation at lower temperatures as a result of a more stable microbial community, emphasizing the advantage of carrier‐based biofilms, with the potential safeguarding of the community to transient unfavorable environmental changes and thereby preventing wash out.

## Conflict of Interest

No conflict of interest is declared.
