# Supplementary material for: The role of inoculum and reactor configuration for microbial community composition and dynamics in mainstream partial nitritation anammox reactors
Source: Microbiologyopen. 2017 Mar 10;6(4):e00456. doi: 10.1002/mbo3.456 (PMC5552961; doi:10.1002/mbo3.456)
Supplement: Supplementary file 1 [file MBO3-6-na-s001.docx]

**Appendix A. Supplementary Information**

Composition of synthetic wastewater, amplicon library preparation information, operating temperature profile, community composition graph at phylum level, PCA plot for the inoculum biomass, Venn diagram for OTU clustering, Hellinger distance and alpha diversity plot, Proteobacteria abundance profile at family level, reactor operational performance parameters, diversity indices table, trends of dominant OTUs dynamics and abundance table of Proteobacteria, Chloroflexi, Chlorobi, Actinobacteria and Acidobacteria at class level.
