# Supplementary material for: The role of inoculum and reactor configuration for microbial community composition and dynamics in mainstream partial nitritation anammox reactors
Source: Microbiologyopen. 2017 Mar 10;6(4):e00456. doi: 10.1002/mbo3.456 (PMC5552961; doi:10.1002/mbo3.456)
Supplement: Supplementary file 2 [file MBO3-6-na-s002.docx]

**Supplementary Information**

**The role of inoculum and reactor configuration for microbial community composition and dynamics in mainstream partial nitritation anammox reactors**

Shelesh Agrawal^1,2*^, Søren M. Karst^3^, Eva M. Gilbert^2,4^, Harald Horn^2^, Per H. Nielsen^3^, Susanne Lackner^1,2^

*^1^* *Technische Universität Darmstadt, Institute IWAR, Chair of Wastewater Technology II, Franziska-Braun-Straße 7,64287 Darmstadt, Germany*

*^2^* *Karlsruhe Institute of Technology, Engler-Bunte-Institut, Chair for Water Chemistry and Water Technology, Engler-Bunte-Ring 1, 76131 Karlsruhe, Germany*

^3^ *Center for Microbial Communities,* *Department of Chemistry and Bioscience, Aalborg University, Aalborg, Denmark*

*^4^EnviroChemie GmbH, In den Leppsteinswiesen 9, 64380 Rossdorf, Germany*

**E-mail:* [*s.agrawal@iwar.tu-darmstadt.de*](mailto:s.agrawal@iwar.tu-darmstadt.de)*, Phone: +49 615 116 21039, Fax: +49 615 116 20305.*

1. Composition of synthetic wastewater used as feed for all four lab reactors.

Synthetic wastewater: 282 mg l^-1^ NH_4_HCO_3_, 11.3 mg 1^-1^ KH_2_PO_4_, 126 mg l^-1^ MgSO_4_•7H_2_O, 75.6 mg l^-1^ CaCl_2_•2H_2_O

Trace elements: 25 µg l^-1^ EDTA, 5 µg l^-1^ FeSO_4_, 0.4 µg l^-1^ ZnSO_4_•7H_2_O, 0.2 µg l^-1^ CoCl•6H_2_O, 1 µg l^-1^ MnCl_2_•4H_2_O, 0.25 µg l^-1^ CuSO_4_•5H_2_O, 0.2 µg l^-1^ NaMoO_4_•2H_2_O, 0.2 µg l^-1^ NiCl_2_•6H_2_O, 0.2 µg l^-1^ NaSeO_4_•10H_2_O and 0.01 µg l^-1^ H_3_BO_4_.

1. Biomass sampling and PCR amplification details, used for amplicon library preparation.

For biomass sampling, 100 mg wet biomass was collected in sterile 2 ml Eppendorf tubes and stored at -80^o^C for further analysis. As a pre-step for biomass sampling, biomass from carriers was completely scrapped off the compartments and homogenized, whereas, for suspended and granular biomass samples were centrifuged. The primer pair (forward primer 515F: 5'-GTGCCAGCMGCCGCGGTAA-3' and reverse primer 806R: 5'-GGACTACHVGGGTWTCTAAT-3') was used. Each PCR reaction was carried out in a total volume of 25 µl containing 2.5 µL 10X HF PCR Buffer (Life Technologies), 2 µL dNTP (5mM), 0.75 µL MgSO4 (50mM), 0.1 µL Platinum Taq DNA Polymerase HF (0.5 U/µL) (Life Technologies), 2 µL of 16S V4 adaptor and 15.65 µL DNA grade H_2_O. The PCR program included activation at 94^o^C for 3 min, followed by 30 cycles, each 94^o^C for 45 sec (denaturation), 50^o^C for 60 sec (annealing), 72^o^C for 90 sec (elongation), and final extension at 72^o^C for 10 min.

1. Microbial Community Composition and Dynamics

Comparing the diversity among the four inocula originating from four different full-scale PNA systems, it was found that overall the inocula of the SBRs and MBBRs were significantly horizontally distant, and the samples of the MBBRs grouped together (S. Fig. 3). The samples of the SBRs showed vertical separation, mainly due to OTU 797, however less significant than the observed horizontal separation from the inocula of the MBBRs.

The SBRs exhibited a significant change compared to the MBBRs (S. Fig. 5, S. Fig. 6). Estimates of the Hellinger dissimilarity and alpha diversity suggested that the magnitude of the community shift was larger in the SBRs than the MBBRs. In the SBRs, the community shift occurred in all phases (S. Fig. 2, S. Fig. 5), especially in SBR1, corresponding to a decrease in reactor performance (Figure 2 and Table 1). This change in community composition in each reactor also related to a decrease in diversity in comparison with the respective inocula and was maximum in SBR1. The resulting Venn diagrams also demonstrated this dynamic OTU overlap patterns for each reactor over time (S. Fig. 4B-D). The relatively consistent OTU overlap pattern amongst the MBBRs further supported higher dynamic community shifts in the SBRs. Although the communities in the MBBRs were relatively stable, minor difference in communities were observed, with more dynamic behavior in MBBR1 (S. Fig. 5). This community change in MBBR1 was observed in all phases.

**Figures:**


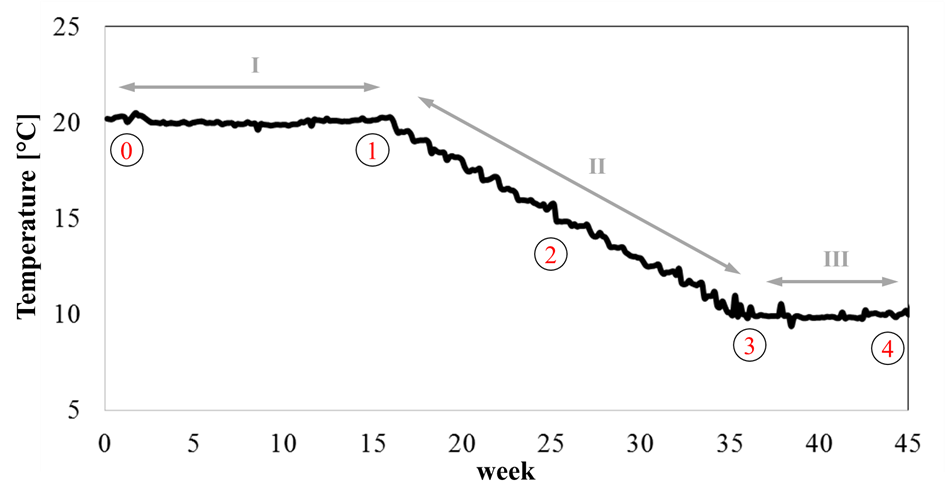


S. Fig. 1: Operating temperature over the experimental period in all reactors. Phase I – operation at stable 20°C; phase II, temperature decrease from 20°C down to 10°C (0.5°C reduction per week), phase III – operation at 10°C. Data points 0 – 4 indicate the conditions at inoculation (0), after operation at 20°C (week 15, point 1), at 15°C (week 25, point 2), at the end of the temperature decrease (10°C, point 3) and after operation at 10°C (week 45, point 4).


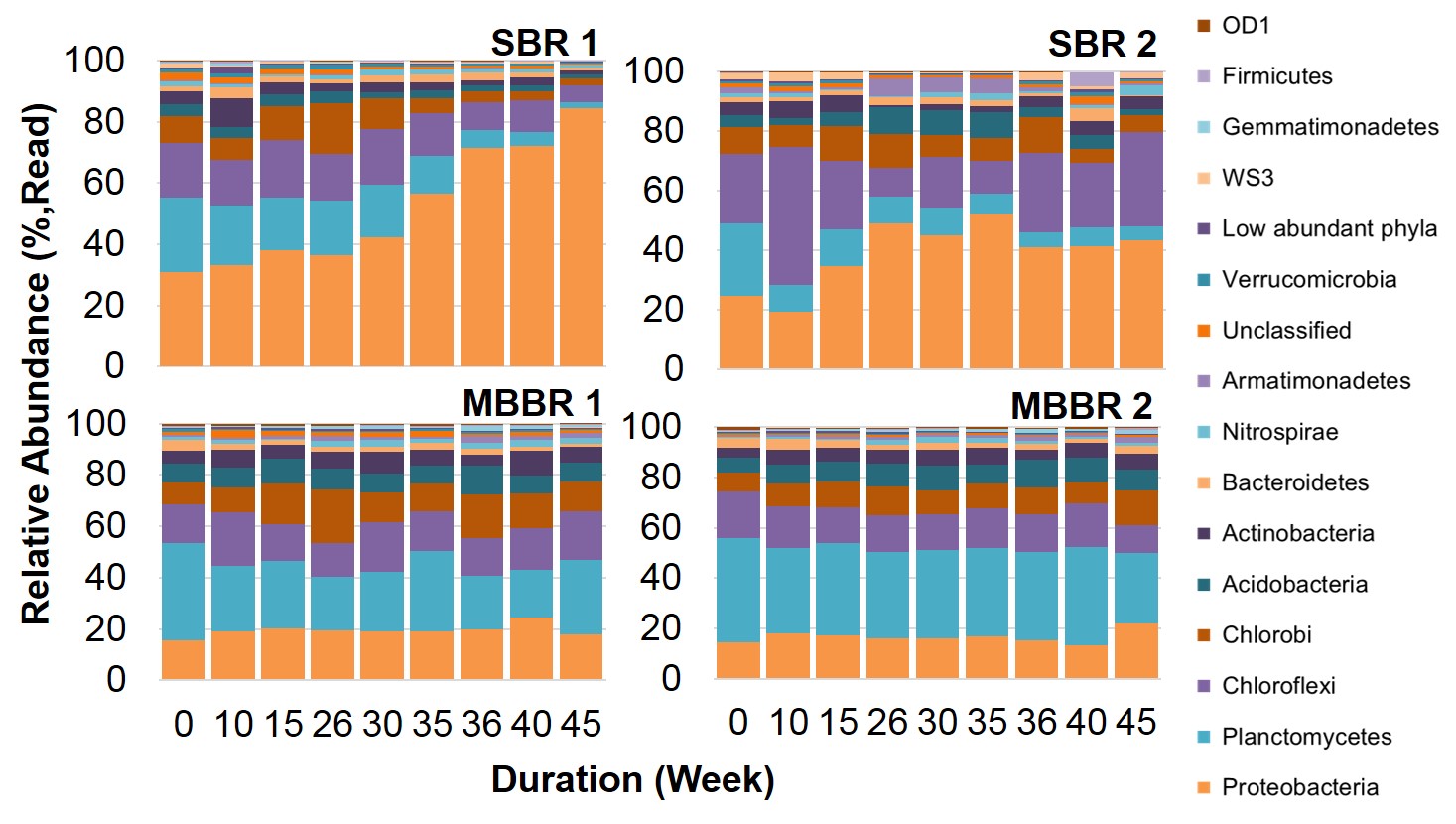


S. Fig. 2: Relative read abundance of phyla in each reactor over the entire operation. SBR1 (suspended biomass), SBR2 (granular biomass), MBBR1 (BiofilmChipTM M) and MBBR2 (K3®). Low abundant phyla include phyla <1% mean abundance across all the samples. Low abundant phyla included BRC1, WPS-2, GN04, Thermi, Caldithrix, Cyanobacteria, GN02, TM6, FBP, NKB19, TM7, Chlamydiae, Spirochaetes, WS2, OP11, SR1, WS6, OP9, WS4, SBR1093, Tenericutes, Fibrobacteres, Lentisphaerae, MVP-21, LD1, WWE1, Synergistetes, Elusimicrobia and Hyd24-12. Colour scheme in bar corresponds to colours representing in legends from bottom to upward.


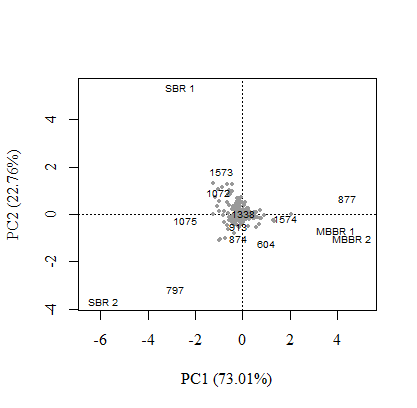


S. Fig. 3: Principal component analysis of the microbial community composition at OTU level for the inoculum biomass of each reactor was performed. Reactor name represents composition profile. OTUs contributing most to the variance between the samples are provided and the rest are represented as grey circles.


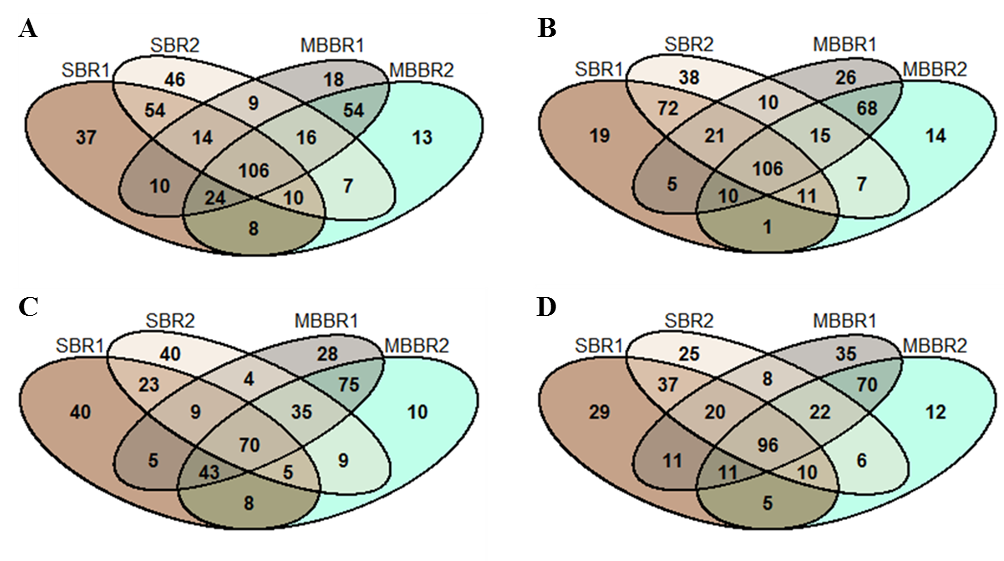


S. Fig. 4: Venn diagrams demonstrating OTU cluster overlap within (A) the inoculum samples; (B) biomass samples from phase I; (C) biomass samples from phase II; (D) biomass samples from phase III. All the samples within each phase were used for the analysis.


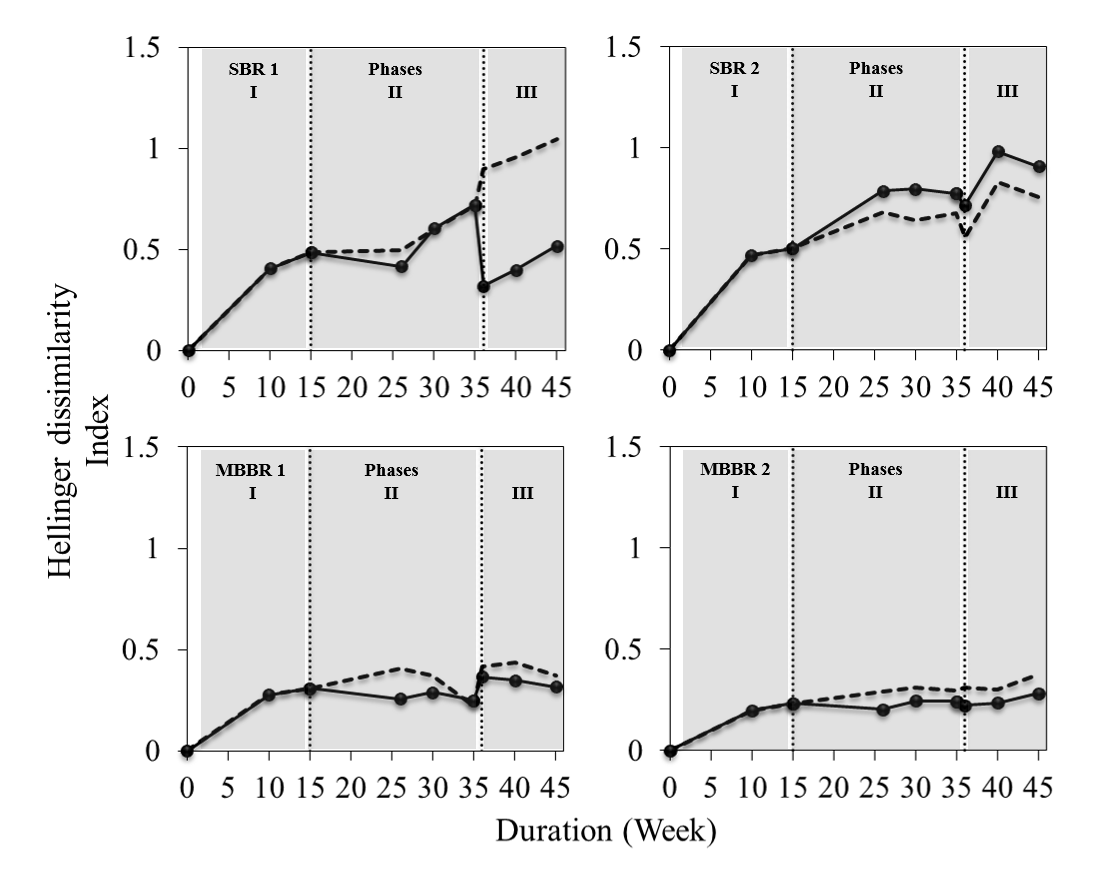


S. Fig. 5: Compositional dissimilarity (Hellinger distance) of the microbial communities in each reactor. Dotted line, represent the dissimilarity of biomass composition over the entire operation in comparison to the inoculum. Solid lines represent the dissimilarity within each operational phase (compared to beginning of the phase).


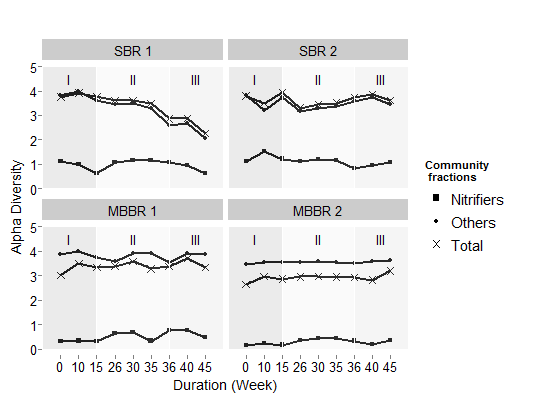


S. Fig. 6: Diversity within each reactor over time was determined by Shannon-Weiner indices. Diversity in each sample is segregated into diversity due to nitrifying community, heterotrophic community and also the total diversity.


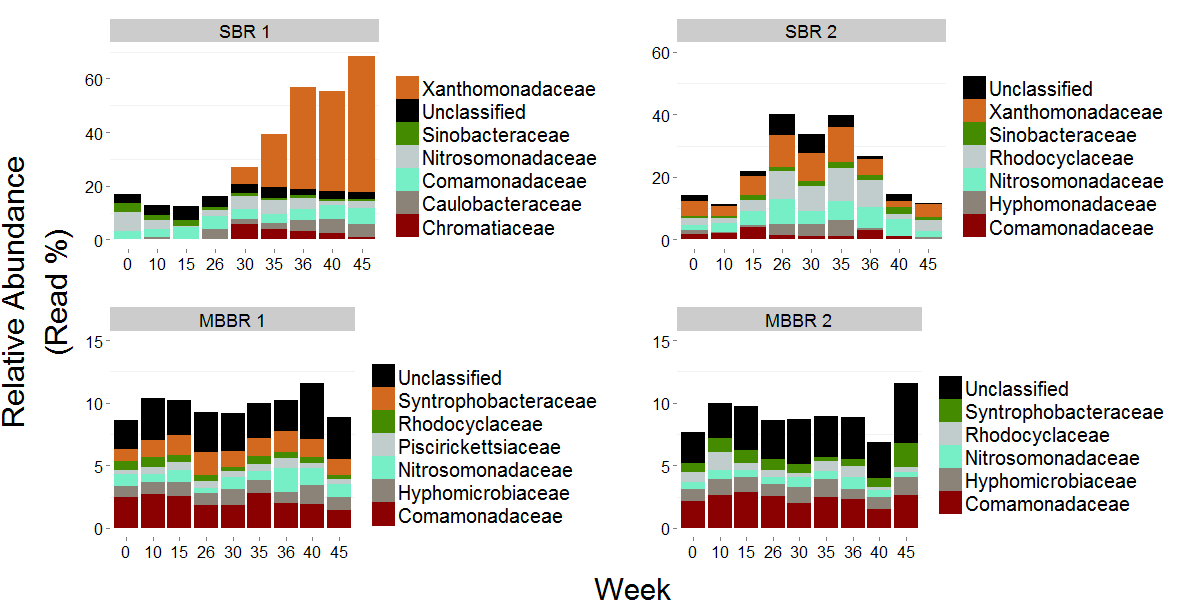


S. Fig. 7: Relative read abundance of top 10 abundant OTUs associated with the families within Proteobacteria for individual reactor (SBR1 (suspended biomass), SBR2 (granular biomass), MBBR1 (BiofilmChipTM M) and MBBR2 (K3®)). Black colour in graphs represents unclassified family within Proteobacteria. Y-axis limits differ for SBR1, SBR2 and MBBR1, MBBR2 to attain better visualization for low abundant Proteobacteria in R2 and R4. Colour scheme in bar corresponds to colours representing in legends from down to upward.

**Tables**

S. Table 1: Reactor operational performance parameters

|  | 20°C | 20°C | 15°C | 10°C | 10°C |
| --- | --- | --- | --- | --- | --- |
| week | 0 | 15 | 25 | 35 | 45 |
| Time Point | 0 | 1 | 2 | 3 | 4 |
|  |  |  | SBR 1 |  |  |
| N_tot_ loading [g-N m^-3^ d^-1^] | 43 ± 11 | 7 ± 2 | 6 ± 1 | 3.2 ± 0.4 | 12 ± 1 |
| NH_4_^+^ removal [%] | 93 ± 4 | 79 ± 7 | 75 ± 15 | 80 ± 7 | 79 ± 13 |
| NO_2_^-^  production [%] | 1 ± 2 | 0.4 ± 0.1 | 40 ± 11 | 62 ± 9 | 61 ± 7 |
| NO_3_^-^  production [%] | 16 ± 3 | 50 ± 14 | 15 ± 4 | 6 ± 3 | 5 ± 5 |
|  |  |  | SBR 2 |  |  |
| N_tot_ loading [g-N m^-3^ d^-1^] | 84 ± 23 | 23 ± 4 | 34 ± 3 | 9.4 ± 1 | 8.5 ± 2.2 |
| NH_4_^+^ removal [%] | 95 ± 5 | 79 ± 13 | 79 ± 7 | 80 ± 12 | 67 ± 12 |
| NO_2_^-^  production [%] | 0.2 ± 0.2 | 1 ± 5 | 0.4 ± 0.1 | 54 ± 13 | 22 ± 24 |
| NO_3_^-^  production [%] | 21 ± 10 | 39 ± 7 | 50 ± 14 | 2.8 ± 3.4 | 6.4 ± 1.6 |
|  |  |  | MBBR 1 |  |  |
| N_tot_ loading [g-N m^-3^ d^-1^] | 210 ± 95 | 15 ± 1 | 64 ± 14 | 25 ± 6 | 25 ± 5 |
| NH_4_^+^ removal [%] | 91 ± 6 | 84 ± 2 | 81 ± 8 | 76 ± 8 | 70 ± 6 |
| NO_2_^-^  production [%] | 0.3 ± 0.2 | 0 ± 0 | 0 ± 0 | 31 ± 10 | 30 ± 10 |
| NO_3_^-^  production [%] | 31 ± 5 | 31 ± 6 | 33 ± 6 | 12 ± 6 | 15 ± 8 |
|  |  |  | MBBR 2 |  |  |
| N_tot_ loading [g-N m^-3^ d^-1^] | 196 ± 95 | 20 ± 2 | 33 ± 11 | 14 ± 3 | 19 ± 1 |
| NH_4_^+^ removal [%] | 97 ± 4 | 84 ± 3 | 85 ± 3 | 92 ± 5 | 95 ± 2 |
| NO_2_^-^  production [%] | 0.8 ± 0.3 | 0 ± 4 | 0.5 ± 0.6 | 4 ± 2 | 4 ± 3 |
| NO_3_^-^  production [%] | 21 ± 9 | 24 ± 7 | 31 ± 8 | 8 ± 6 | 11 ± 6 |

S. Table 2: Diversity indices of all four reactors at end of different operational phases.

| **Reactor** | **Phase** | **Week** | **H index^1^** | **Evenness** | **Richness^2^** |
| --- | --- | --- | --- | --- | --- |
| **SBR 1** | **I** | **0** | 4.38 | 0.72 | 446 |
|  |  | **15** | 4.41 | 0.73 | 410 |
|  | **II** | **35** | 4.04 | 0.69 | 356 |
|  | **III** | **45** | 2.83 | 0.49 | 335 |
| **SBR 2** | **I** | **0** | 4.34 | 0.74 | 366 |
|  |  | **15** | 4.52 | 0.74 | 446 |
|  | **II** | **35** | 4.00 | 0.70 | 293 |
|  | **III** | **45** | 4.20 | 0.70 | 396 |
| **MBBR 1** | **I** | **0** | 3.62 | 0.61 | 385 |
|  |  | **15** | 3.95 | 0.65 | 454 |
|  | **II** | **35** | 3.96 | 0.65 | 447 |
|  | **III** | **45** | 3.93 | 0.65 | 409 |
| **MBBR 2** | **I** | **0** | 3.21 | 0.55 | 357 |
|  |  | **15** | 3.42 | 0.57 | 410 |
|  | **II** | **35** | 3.46 | 0.59 | 352 |
|  | **III** | **45** | 3.73 | 0.63 | 380 |

^1^ Shannon-Weiner index; ^2^ No. of OTUs detected. Diversity estimation was performed at cutoff value of sequence reads >10. SBR1, Suspended biomass; SBR2, Granular biomass; MBBR 1, BiofilmChip^TM^ M; and MBBR 2, K3^®^

S. Table 3: Dynamics of the most dominant OTUs over time in all the four reactor.


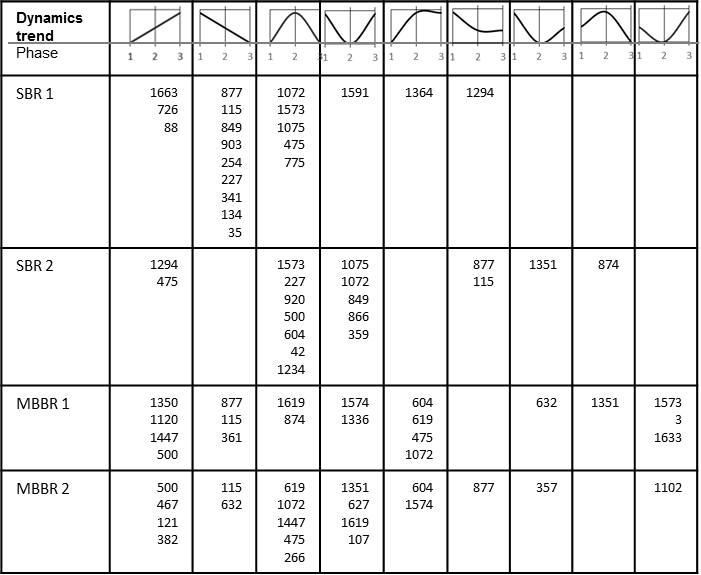


S.Table 4: Relative read abundance (in percentage of total reads) at class level of Proteobacteria, Chloroflexi, Chlorobi, Actinobacteria and Acidobacteria. The table shows the read abundance of biomass at different phase of operation (i.e. week 0, initial biomass; week 15, at 20^o^C; week 36, at 10^o^C; week 45, at 10^o^C i.e. at the end of operation).

| **Reactor** | **SBR1** | | | |  | **SBR2** | | | | |
| --- | --- | --- | --- | --- | --- | --- | --- | --- | --- | --- |
| **Week Class** | **0** | **15** | **36** | **45** |  | **0** | | **15** | **36** | **45** |
| **Proteobacteria** | **30.93** | **37.92** | **71.49** | **84.51** |  | **24.83** | **34.91** | | **41.1** | **43.36** |
| Betaproteobacteria | 14.79 | 16.53 | 15.26 | 15.95 |  | 11.18 | 18.18 | | 25.01 | 14.16 |
| Gammaproteobacteria | 8.43 | 8.86 | 46.42 | 57.33 |  | 4.99 | 5.32 | | 4.02 | 15.91 |
| Alphaproteobacteria | 5.83 | 10.49 | 9.33 | 10.48 |  | 6.21 | 8.73 | | 9.62 | 11.46 |
| Deltaproteobacteria | 1.81 | 1.93 | 0.37 | 0.44 |  | 2.44 | 2.66 | | 2.44 | 1.81 |
| TA18 | 0.04 | 0.07 | 0.03 | 0.06 |  | 0.01 | 0.02 | | 0.01 | 0.01 |
| Unclassified | 0.03 | 0.04 | 0.08 | 0.25 |  | 0 | 0 | | 0 | 0.01 |
| Epsilonproteobacteria | 0 | 0 | 0 | 0 |  | 0 | 0 | | 0 | 0 |
|  |  |  |  |  |  |  |  | |  |  |
| **Chloroflexi** | **18.05** | **18.96** | **9.12** | **5.56** |  | **23.11** | **23.18** | | **26.54** | **31.42** |
| Anaerolineae | 10.58 | 10.15 | 6 | 3.11 |  | 19.14 | 17 | | 23.25 | 26.54 |
| Thermomicrobia | 1.73 | 2.35 | 1.15 | 0.69 |  | 1.63 | 2 | | 1.15 | 1.69 |
| TK17 | 4.41 | 4.74 | 1.35 | 1.36 |  | 1.41 | 2.49 | | 1.34 | 1.8 |
| Ellin6529 | 0.59 | 0.73 | 0.23 | 0.14 |  | 0.42 | 0.82 | | 0.32 | 0.79 |
| S085 | 0.13 | 0.33 | 0.08 | 0.05 |  | 0.11 | 0.22 | | 0.12 | 0.14 |
| Unclassified | 0.34 | 0.52 | 0.24 | 0.1 |  | 0.26 | 0.44 | | 0.19 | 0.3 |
| Gitt-GS-136 | 0.04 | 0.03 | 0.01 | 0 |  | 0.02 | 0.1 | | 0.04 | 0.05 |
| TK10 | 0.21 | 0.1 | 0.06 | 0.1 |  | 0.12 | 0.11 | | 0.1 | 0.11 |
| Chloroflexi | 0.02 | 0.01 | 0 | 0.01 |  | 0 | 0 | | 0.03 | 0 |
| SHA-26 | 0 | 0 | 0 | 0 |  | 0 | 0 | | 0 | 0 |
|  |  |  |  |  |  |  |  | |  |  |
| **Chlorobi** | **8.75** | **11.17** | **3.68** | **2.14** |  | **9.15** | **11.68** | | **11.96** | **5.6** |
| Ignavibacteria | 6.69 | 10.53 | 2.76 | 1.57 |  | 5.99 | 10.54 | | 10.15 | 4.31 |
| SJA-28 | 0.59 | 0.48 | 0.45 | 0.29 |  | 2.32 | 0.72 | | 1.19 | 0.94 |
| OPB56 | 0.92 | 0.02 | 0.4 | 0.22 |  | 0.53 | 0.08 | | 0.16 | 0.04 |
| BSV26 | 0.55 | 0.14 | 0.07 | 0.06 |  | 0.31 | 0.34 | | 0.46 | 0.31 |
|  |  |  |  |  |  |  |  | |  |  |
| **Acidobacteria** | **3.68** | **3.8** | **1.98** | **1.2** |  | **4.01** | **4.43** | | **3.37** | **2.16** |
| Chloracidobacteria | 1.04 | 0.11 | 0.04 | 0.05 |  | 1.35 | 0.87 | | 0.84 | 0.47 |
| Acidobacteria-6 | 1.19 | 2.35 | 1.09 | 0.83 |  | 1.14 | 2.16 | | 1.41 | 1.32 |
| Solibacteres | 1.33 | 0.98 | 0.73 | 0.28 |  | 0.98 | 0.84 | | 0.77 | 0.09 |
| Unclassified | 0 | 0.01 | 0 | 0 |  | 0.34 | 0.23 | | 0.14 | 0.17 |
| Sva0725 | 0.05 | 0.2 | 0.05 | 0.01 |  | 0.14 | 0.2 | | 0.06 | 0.05 |
| iii1-8 | 0.03 | 0.08 | 0.02 | 0 |  | 0.04 | 0.03 | | 0.08 | 0.01 |
| Acidobacteriia | 0.02 | 0.05 | 0.05 | 0.03 |  | 0.02 | 0.1 | | 0.07 | 0.05 |
| BPC102 | 0.02 | 0.02 | 0 | 0 |  | 0 | 0 | | 0 | 0 |
| DA052 | 0 | 0 | 0 | 0 |  | 0 | 0 | | 0 | 0 |
|  |  |  |  |  |  |  |  | |  |  |
| **Actinobacteria** | **4.47** | **3.89** | **1.47** | **1.4** |  | **4.35** | **5.69** | | **3.79** | **4.36** |
| Thermoleophilia | 1.87 | 1.79 | 0.31 | 0.44 |  | 2.13 | 2.67 | | 1.65 | 2.04 |
| Acidimicrobiia | 2.15 | 1.87 | 0.87 | 0.77 |  | 2.18 | 2.94 | | 2.04 | 2.21 |
| KIST-JJY010 | 0.03 | 0 | 0 | 0 |  | 0 | 0 | | 0 | 0.01 |
| Unclassified | 0.01 | 0 | 0 | 0 |  | 0 | 0 | | 0 | 0 |
| Actinobacteria | 0.4 | 0.23 | 0.29 | 0.18 |  | 0.04 | 0.07 | | 0.09 | 0.1 |
| MB-A2-108 | 0.01 | 0 | 0 | 0.01 |  | 0 | 0 | | 0 | 0 |

| **Reactor** | **MBBR1** | | | |  | **MBBR2** | | | |
| --- | --- | --- | --- | --- | --- | --- | --- | --- | --- |
| **Week Class** | **0** | **15** | **36** | **45** |  | **0** | **15** | **36** | **45** |
| **Proteobacteria** | **15.79** | **20.3** | **19.99** | **18** |  | **14.66** | **17.31** | **15.44** | **22.1** |
| Betaproteobacteria | 6.96 | 8.86 | 8.89 | 8.01 |  | 6.47 | 7.61 | 7.61 | 8.61 |
| Gammaproteobacteria | 2.06 | 3.13 | 3.11 | 2.52 |  | 1.87 | 2.86 | 2.16 | 3.58 |
| Alphaproteobacteria | 4.32 | 5 | 4.4 | 5.01 |  | 4.36 | 4.39 | 4 | 6.46 |
| Deltaproteobacteria | 2.38 | 3.21 | 3.54 | 2.4 |  | 1.91 | 2.41 | 1.63 | 3.42 |
| TA18 | 0.07 | 0.08 | 0.05 | 0.07 |  | 0.03 | 0.02 | 0.02 | 0.03 |
| Unclassified | 0 | 0.02 | 0 | 0.01 |  | 0.02 | 0.02 | 0.02 | 0 |
| Epsilonproteobacteria | 0 | 0 | 0 | 0 |  | 0 | 0 | 0 | 0 |
|  |  |  |  |  |  |  |  |  |  |
| **Chloroflexi** | **14.93** | **14.3** | **14.54** | **18.9** |  | **18.23** | **14.36** | **15.2** | **11.1** |
| Anaerolineae | 10.87 | 9.58 | 11.07 | 13.93 |  | 15.49 | 11.88 | 12.91 | 8.72 |
| Thermomicrobia | 2.22 | 2.35 | 2.31 | 2.83 |  | 1.7 | 1.55 | 1.49 | 1.38 |
| TK17 | 0.52 | 0.58 | 0.36 | 0.65 |  | 0.42 | 0.35 | 0.23 | 0.41 |
| Ellin6529 | 0.47 | 0.51 | 0.36 | 0.44 |  | 0.29 | 0.25 | 0.23 | 0.13 |
| S085 | 0.28 | 0.65 | 0.19 | 0.39 |  | 0.1 | 0.12 | 0.12 | 0.17 |
| Unclassified | 0.19 | 0.2 | 0.08 | 0.21 |  | 0.05 | 0.09 | 0.06 | 0.07 |
| Gitt-GS-136 | 0.21 | 0.2 | 0.06 | 0.19 |  | 0.08 | 0.03 | 0.06 | 0.06 |
| TK10 | 0.12 | 0.1 | 0.01 | 0.16 |  | 0.05 | 0.08 | 0.03 | 0.07 |
| Chloroflexi | 0.04 | 0.06 | 0.08 | 0.04 |  | 0.05 | 0.01 | 0.07 | 0.05 |
| SHA-26 | 0.01 | 0.07 | 0.02 | 0.01 |  | 0 | 0 | 0 | 0 |
|  |  |  |  |  |  |  |  |  |  |
| **Chlorobi** | **8.31** | **15.94** | **17.17** | **11.6** |  | **7.52** | **10.01** | **10.54** | **13.5** |
| Ignavibacteria | 3.34 | 4.35 | 5.24 | 3.61 |  | 2.17 | 3.41 | 3.25 | 3.99 |
| SJA-28 | 4.47 | 11.1 | 11.68 | 7.53 |  | 4.77 | 6.03 | 6.88 | 9.03 |
| OPB56 | 0.35 | 0.29 | 0.15 | 0.15 |  | 0.57 | 0.53 | 0.37 | 0.43 |
| BSV26 | 0.15 | 0.2 | 0.1 | 0.27 |  | 0.01 | 0.04 | 0.04 | 0.06 |
|  |  |  |  |  |  |  |  |  |  |
| **Acidobacteria** | **7.45** | **9.71** | **11.08** | **7.46** |  | **6.13** | **8.24** | **10.84** | **8.3** |
| Chloracidobacteria | 5.48 | 7.24 | 9.36 | 5.27 |  | 4.16 | 6.15 | 9.36 | 6.17 |
| Acidobacteria-6 | 0.94 | 1.34 | 0.81 | 1.54 |  | 1.09 | 1.27 | 0.78 | 1.2 |
| Solibacteres | 1 | 0.95 | 0.84 | 0.56 |  | 0.88 | 0.79 | 0.63 | 0.91 |
| Unclassified | 0 | 0 | 0 | 0 |  | 0 | 0 | 0 | 0 |
| Sva0725 | 0 | 0.02 | 0 | 0.02 |  | 0 | 0 | 0 | 0 |
| iii1-8 | 0.02 | 0.1 | 0.07 | 0.03 |  | 0 | 0.02 | 0.06 | 0.02 |
| Acidobacteriia | 0.01 | 0.02 | 0 | 0.02 |  | 0 | 0 | 0 | 0 |
| BPC102 | 0 | 0.04 | 0 | 0.02 |  | 0 | 0.01 | 0.01 | 0 |
| DA052 | 0 | 0 | 0 | 0 |  | 0 | 0 | 0 | 0 |
|  |  |  |  |  |  |  |  |  |  |
| **Actinobacteria** | **5.06** | **5.41** | **4.43** | **6.02** |  | **4.03** | **5.53** | **3.95** | **6.61** |
| Thermoleophilia | 1.39 | 1.23 | 0.93 | 1.77 |  | 1.21 | 1.64 | 1.27 | 1.54 |
| Acidimicrobiia | 0.96 | 0.77 | 0.94 | 0.83 |  | 0.8 | 0.94 | 0.7 | 0.95 |
| KIST-JJY010 | 1.95 | 2.48 | 2.02 | 2.43 |  | 1.29 | 1.74 | 1.41 | 2.56 |
| Unclassified | 0.42 | 0.63 | 0.29 | 0.6 |  | 0.6 | 0.97 | 0.49 | 1.24 |
| Actinobacteria | 0.34 | 0.3 | 0.25 | 0.39 |  | 0.13 | 0.24 | 0.08 | 0.32 |
| MB-A2-108 | 0 | 0 | 0 | 0 |  | 0 | 0 | 0 | 0 |
